# Report of the Ohio State Dental Examining Board

**Published:** 1885-12

**Authors:** E. G. Betty


### Report of the Ohio State Dental Examining Board.

This Board met in annual session at 12 m., in room No. 3, of the Valley House, at Chillicothe, October 28th, 1885. All members were present excepting Dr. I. Williams, who was incapable of attending on account of serious illness.

The following gentlemen presented themselves for examination:

Clayton H. Davis, Orville, Oswego County, N. Y.; Jas. O. Howkins, Welston, Jackson County, Ohio; Oliver S. Rightmire, Cincinnati, Hamilton County, Ohio.

The Board held several meetings, on Wednesday, Thursday and Friday, during which the examination progressed. On Friday, October 30th, the final meeting was held, four members being present. Dr. C. R. Butler having been re-elected, and Dr. E. G. Betty, being elected to succeed Dr. I. Williams. The organization resulted in the election of Dr. J. Taft, President; Dr. E. G. Betty, Secretary and Treasurer.

The examination of the candidates, both by writing and orally was completed, and being satisfactory, a separate vote was taken upon each candidate, certificates being granted to all.

Adjourned to meet at the Boody House, in Toledo, last Tuesday of October, 1886, at 12 o'clock, noon.

E. G. BETTY, Secretary and Treasurer.

